# Development and acceptability of a brief, evidence-based Dementia Awareness for Caregivers course in low- and middle-income countries

**DOI:** 10.1177/14713012211055316

**Published:** 2021-12-09

**Authors:** Charlotte R Stoner, Monisha Lakshminarayanan, Daniel C Mograbi, Sridhar Vaitheswaran, Elodie Bertrand, Paula Schimidt Brum, Helen Durgante, Cleusa P Ferri, Sarah Mkenda, Richard Walker, Catherine Dotchin, Stella-Maria Paddick, Mina Chandra, Murali Krishna, Bharath Du, Kunnukattil S Shaji, Emily Fisher, Aimee Spector

**Affiliations:** 4918 Centre for Chronic Illness and Ageing, Institute for Lifecourse Development, School of Human Sciences, University of Greenwich, London, UK; 29868 Dementia Care in Schizophrenia Research Foundation (DEMCARES in SCARF), Chennai, Tamil Nadu, India; Department of Psychology, 28099 Pontifical Catholic University of Rio de Janeiro, Brazil; Institute of Psychiatry, Psychology and Neuroscience, King’s College London, UK; 29868 Dementia Care in Schizophrenia Research Foundation (DEMCARES in SCARF), Chennai, Tamil Nadu, India; 555089 Institut de Psychologie, Université de Paris, France; Department of Psychiatry, 28105 Universidade Federal de São Paulo, Brazil; Psychology Department, 28124 Universidade Federal do Rio Grande (FURG), Rio de Janeiro, Brazil; Department of Psychiatry, 28105 Universidade Federal de São Paulo, Brazil; Health Technology Assessment Unit, Hospital Alemão Oswaldo Cruz, Brazil; Occupational Therapy Department, Kilimanjaro Christian Medical University College, Tanzania; Population Health Sciences Institute, 5994 Newcastle University, UK; Population Health Sciences Institute, Newcastle University, UK; North Tyneside General Hospital, 6072 Northumbria Healthcare National Health Service Foundation Trust, UK; Clinical and Translational Medicine, 5994 Newcastle University, Tyne and Wear, UK; Gateshead Health NHS Foundation Trust, UK; Centre of Excellence in Mental Health, 575015 Atal Bihari Vajpayee Institute of Medical Sciences (formerly Post Graduate Institute of Medical Education and Research) and Dr Ram Manohar Lohia Hospital, India; Foundation for Research and Advocacy in Mental Health (FRAMe), Mysore, India; Foundation for Research and Advocacy in Mental Health (FRAMe), Mysore, India; Viveka Hospital, Mysore, India; Kerala University of Health Sciences, Thrissur, Kerala, India; Research Department of Clinical, Educational and Health Psychology, 4919 University College London (UCL), London, UK

**Keywords:** Education, dementia, intervention, caregiving, stigma

## Abstract

**Background:**

Knowledge of and attitudes towards dementia vary across countries, and for caregivers in low- and middle-income countries (LMICs), access to information can be challenging. There is an urgent need for brief, easily accessible and culturally appropriate educational courses for caregivers of persons with dementia, providing much needed information whilst addressing important psychological concepts such as stigma.

**Methods:**

An international and multidisciplinary team developed Dementia Awareness for Caregivers (DAC) courses in four stages: (1) scoping review and module agreement, (2) development of an International template (DAC-International) containing a standardised process for adding information, (3) development of local DACs using a standardised format and (4) acceptability of courses in Brazil, India and Tanzania.

**Findings:**

The DAC-International was developed, comprising three modules: ‘What is dementia?’; ‘Positive engagement’ and ‘Caring for someone with dementia’. Three local versions were developed from this (DAC-Brazil, DAC-India and DAC-Tanzania), where additions of country-specific information included prevalent stereotypes and the addition of culturally relevant case studies. An initial field test was conducted in each country (*n* = 85), which indicated acceptability to participants.

**Conclusions:**

The methods used here resulted in culturally valid and acceptable educational courses for carers of people with dementia. Future work will consist of large-scale, formal evaluations and the development of additional local courses.

A global health approach to dementia is of increasing importance, with the World Health Organisation (WHO) adopting a worldwide plan that calls on governments to meet targets for the advancement of awareness, risk reduction, diagnosis, care and treatment, support for care partners and research (WHO, 2017). However, in practice, countries are vastly diverse with differing knowledge of, and attitudes towards people with dementia, healthcare systems and treatment options (Alzheimer’s Disease International [ADI], 2015; 2019).

For family caregivers, access to information and support can vary widely. Many educational courses exist but, predominantly, these have been developed for use in Western, high income countries (HIC) and adapted for use in other countries (Hinton et al., 2019). The process of adaptation means cross-culturally valid information is delivered, but methods for adaptation work can also differ significantly with no internationally agreed methods. The use of a framework or template for which country specific information can be added may be a more appropriate and cross-culturally valid means of increasing knowledge and awareness of dementia.

Caring for someone living with dementia is complex and can be influenced by knowledge of dementia, stigma and burden (Kahn et al., 2016). Further highlighting the complexity of these concepts, even where stigma is low, attitudes towards persons with dementia can still be problematic, with a recent global survey documenting that, whilst 91% (*n* = 60,860) of respondents stated that no one should have to hide a diagnosis of dementia, 85% of respondents living with dementia reported that their opinions had not been taken seriously (ADI, 2019). In the same survey, carers expressed positive aspects of caregiving, but the findings highlighted a clear need for support and information to overcome challenges in the role.

Dementia in low- and middle-income countries (LMICs) accounts for 63% of global cases (Vaitheswaran & Ramanujam, 2018). There are also countries where, sometimes, awareness can be low and stigma high, and there is a need to deliver educational courses that address these issues. In order to target both of these concepts, a multi-component course underpinned by psychological theories and practice is needed. However, educational courses in LMICs can differ from those delivered in HICs, with those delivered in LMICs more likely to omit psychosocial components such as communication training, resilience building and managing stigma (Hinton et al., 2019).

Some interventions incorporate principles which are considered key for effective communication and interaction for people with dementia. The most evidence-based example is Cognitive Stimulation Therapy (CST), an effective, low-cost (Knapp et al., 2006) psychosocial intervention demonstrated to improve cognition and quality of life (Spector et al., 2003). It was also found to be most effective and implementation ready among other psychosocial interventions delivered in LMICs (Stoner et al., 2021). It can be culturally adapted (Aguirre et al., 2014) and is implemented globally. There is evidence for its effectiveness in LMICs (e.g. Paddick et al., 2017) and one of the core intervention components of CST is the underlying and universal 18 psychological principles on which it is based. In particular, principles of promoting mental stimulation, encouraging new thoughts, ideas and associations, social inclusion, involvement and respect may promote a conducive atmosphere where learning is encouraged in a supportive environment. As such, a training course that satisfies the need for information and practical advice, whilst incorporating the biopsychosocial principles of CST may result in a similarly conducive learning environment for a caregiver or supportive other and an improved home environment for a person with dementia.

## Aims

1. To develop a brief, international dementia awareness course suitable for use as a template.
2. To add country specific information to the international template, thereby creating local dementia awareness courses for use in three diverse LMICs: Brazil, India and Tanzania.
3. To evaluate the acceptability of the adapted international template for each country through field testing.

## Method

**Figure 1.**
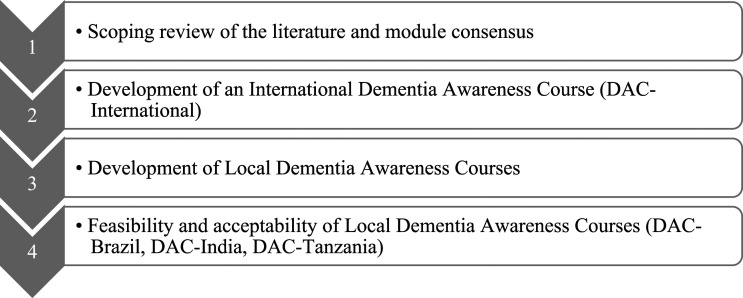
Methodology overview.

The Dementia Awareness Course (DAC) was developed as part of the ongoing CST-International programme to meet a secondary objective of increasing awareness and skills in the management of dementia for caregivers and families (Spector et al., 2019) in Brazil, India and Tanzania. The course was developed iteratively, with continual feedback from researchers, clinicians and patient and public involvement (PPI). First, we synthesised the literature on caregiver interventions around the world and in LMICs, and modules for the course were decided upon. Second, an international version of the course was developed and agreed upon. Third, representatives from Brazil, India and Tanzania amended the international version to a local version and sought PPI feedback. Finally, the course was delivered to a small number of family caregivers in each setting and feedback on acceptability was sought (Figure 1).

### Stage 1: Scoping review of the literature and module consensus

Initial evidence was sought from all countries using a scoping review of the literature via Google Scholar. Search terms used were broad and included ‘training’ OR ‘education’ AND ‘carer’ AND ‘dementia’. Variations of these terms were used as appropriate and no cut off dates were specified. In addition, both professional and family caregiver interventions were initially sought. To ensure breadth of evidence, systematic reviews were prioritised. Evidence from around the world was then combined with a more specific search of caregiver interventions in LMICs, with a focus on educational or training programmes on dementia. For this search, modules and course content were sought and examined to determine relevance across countries.

The literature sourced was then examined for content coverage, length and outcomes. Particular attention was paid to courses that complemented any of the 18 CST principles, resulted in significant outcomes and were used in LMICs. This combined with the clinical experience of researchers in Brazil, India, Tanzania and the United Kingdom (UK) led to an initial set of modules being proposed. These modules were then discussed, with consensus sought. Amendments were made where appropriate and a final set of modules were agreed upon.

### Stage 2: Development of the international version

To ensure the underpinning theories remained consistent across countries but content could be added for specific settings, one international version of the course was developed and consisted of a word document Course Guide and accompanying PowerPoint presentation. Feedback was sought on both documents from the international team and amendments were made, as necessary. This resulted in a DAC-International template that could be amended for local settings.

### Stage 3: Development of local versions

Following consensus on the DAC-International, representatives from Brazil, India and Tanzania were asked to make standardised local additions and adaptations. These additions included country specific information, lacking from the international template version of the course. Representatives were asked to ensure that additions were made to account for healthcare systems, geographical and cultural differences. Representatives were also instructed to obtain PPI feedback, in particular from at least two family caregivers, after making their local additions. The course was then translated for each setting, using either professional translators or bilingual researchers. Translation aimed to ensure both semantic and conceptual equivalency of terms used. The final version of the DAC in each setting was reviewed by the international team, and if no further recommendations were made by the team, the local versions were approved.

### Stage 4: Local field testing to assess acceptability

The local version of the courses was piloted with family caregivers whose relative or friend with dementia was receiving CST as part of the CST-International research programme. This was deemed appropriate as it would allow facilitators access to small groups of caregivers, who could receive the course whilst the person with dementia was cared for in CST. Furthermore, by running the course concurrently to an intervention for people with dementia, direct and indirect costs were kept to a minimum. Additional recruitment took place through local caregiver associations, outpatient clinics and over social media. The course was delivered by the representative who led on the local additions and caregivers were encouraged to share their experiences throughout. Dedicated time was left following completion of the material for caregivers to provide formal written feedback on the course.

The aim of stage 4 was to establish acceptability of content through stakeholder engagement. The evaluation was not a formal research study, no standardised outcome measures were used, and no personal or patient information was collected from attendees. Therefore, consent for research was not required for this stakeholder engagement activity (Health Research Authority / INVOLVE, 2016). To ascertain acceptability, voluntary and informal feedback was elicited using questions developed by the international team. The following questions were given to all attendees to complete following the course:

1. What did you hope to learn/achieve from today’s course? Did you achieve this?
2. What was the most helpful part of the day?
3. What was the least helpful part of the day?
4. Is there anything we did not cover that you think should be included?
5. Would you recommend this course? Why?

## Findings

### Stage 1: Scoping review of the literature and module consensus

Educational interventions for family caregivers were assessed in a meta-analysis of seven RCTs with 764 informal caregivers in the community (Jensen et al., 2015). The interventions took place in France, Russia, Peru, the United States of America (USA), Austria, Switzerland, Spain and Iran. All trials examined the effects of a structured education programme devised to cover a set of pre-defined topics relevant to dementia caring and the caregiver role, specific to the country in question. Three trials were one-to-one based education, and four evaluated group-based education programmes. The total duration of education ranged from two and a half to 24 hours with an average duration of about 10 hours. In all studies, the interventions were delivered by health professionals. Primary outcomes were quality of life and caregiver burden, but transition to long-term care and caregiver depression were also reported. For studies that measured burden, educational programmes had a moderate effect (SMD = 0.52; 95% confidence interval (CI) 0.79 to 0.26; *I*^2^ = 40%). Shorter programmes were more effective than longer programmes (*p* = 0.03) for burden. This was especially true for LMICs (Peru and Russia) where the effect on caregiver burden was more than in HICs. The authors hypothesised that this may have been due to a lower standard of treatment as usual in these countries. Findings for quality of life were mixed, with no meta-analysis conducted. There was a small effect of educational programmes on caregiver depression at 6 months (SMD = 0.37; 95% CI 0.65 to 0.09; *I*^2^ = 0%).

In LMICs and captured in the previous systematic review, the 10/66 ‘Helping Carers to Care’ intervention was the most researched. Originally developed in India (Prince & Group, 2004) but designed to be delivered internationally, the original intervention consisted of three modules: assessment (one session), basic education (two sessions) and training on problem behaviours (two sessions). The overall aim of this intervention was to reduce burden and psychological stress and increase quality of life for the caregiver, whilst increasing quality of life and reducing Behavioural and Psychological Symptoms of Dementia (BPSD) for people with dementia. In the original trial in Goa, India, 59 participants and their caregivers completed the programme resulting in a significant decrease in mental health problems and distress caused by neuropsychiatric behaviours. There was no significant effect on burden, activities of daily living for the person with dementia or neuropsychiatric symptoms for the person living with dementia.

A second Helping Carers to Care study in a LMIC was also identified, describing 56 dyads in Peru (27 interventional and 29 waiting list controls), who completed the intervention. Results were similar with caregivers in the intervention group seeing a decrease in burden whilst this was increased in the control group. However, quality of life for caregivers decreased in both groups, although to a lesser extent in the intervention group, and there were no significant differences in BPSD for both groups. Quality of life for people with dementia was only assessed in approximately one-third of participants for which there was a non-significant increase in the intervention group and a non-significant decrease in the control group (Guerra et al., 2011).

As the Helping Carers to Care intervention is a train-the-trainer programme requiring 2 days of training and five weekly 30 minute sessions, it was deemed too intensive for the purposes of the current study. Furthermore, in the first module ‘dementia’, the information presented was often presented from a problem oriented perspective, focussing on managing behavioural aspects of dementia including repeated questioning, aggression and personal hygiene (ADI, 2009). Whilst this is undoubtedly an important area for family caregivers, well-evidenced psychological theories and frameworks such as excess disability (Brody, 1971) and the biopsychosocial model of dementia (Spector & Orrell, 2010) were missing.

A consensus meeting was convened in Chennai (India) involving 13 experts (seven psychiatrists, one researcher and five psychologists). The existing literature was appraised, and participants were asked for their ideas on what a brief course for family caregivers should contain. A list of topics that all attendees found relevant for such a course was generated. Some recurrent themes were

1. Understanding dementia;
2. Addressing stigma and misconceptions around dementia;
3. Improving communication between the caregiver and the person with dementia;
4. Practical ways to care for someone with dementia (personal care, feeding, hygiene, behavioural symptoms, managing risk etc.);
5. Coping with the stress and burnout that comes with caring for someone.

Based on this, three initial modules were proposed: ‘What is dementia?’; ‘Interventions’ and ‘Positive Engagement’ (Table 1).

**Table 1.** Initial modules proposed.

	Module 1: What is dementia?	Module 2: Interventions	Module 3: Positive engagement
Time	30 min	60 min	90 min
Delivery	Lecture	Lecture and case studies	Lecture and activities
Topics	Types of dementia, biopsychosocial model, prevalence of older adults, prevalence of dementia, common myths and stages of dementia	Interventions for cognition, stress and distress, activities of daily living, risk management, impact on caregivers, carer needs, peer support, knowledge support, counselling and government/healthcare support	Consistent engagement, new thoughts, ideas and associations, implicit learning, stimulating language, stimulating executive function, person centred activities, fun, sensitive orientation, opinions, cues for recall, maximising potential, reminiscence, respect, involvement, choice, inclusion and relationships

This format was discussed during the meeting and it was agreed that module three was too information intensive. Furthermore, there was an overriding focus on psychological concepts and, as such, practical information and advice for caregivers was missing from the course as a whole. Module 3 was subsequently re-formulated into ‘Caring for someone with dementia’ and practical information on topics such as nutrition, medications and interventions were combined with psychological concepts such as caregiver needs. Additionally, the format of all modules was changed to include both activities and discussion groups as well as lectures. As such, the finalised modules were ‘What is dementia?’, ‘Positive engagement’ and ‘Caring for someone with dementia’ (Table 2). The modules were preceded by a five-minute welcome and introductions and followed by 10-min feedback and reflection time.

**Table 2.** Finalised modules.

	Module 1: What is dementia?	Module 2: Positive engagement	Module 3: Caring for someone with dementia
Time	25 min	90 min	80 min
Delivery	Lecture and activity	Lecture, case studies, activities	Lecture and activities
Topics	background on dementia, dementia in country in question, types, dementia progression and models of dementia	Personhood, malignant social psychology, positive person work, psychological needs, mental stimulation, new thoughts, ideas and associations, promoting strengths and mitigating weaknesses, opinions rather than facts, maximising potential, positive communication, involvement and inclusion, relationships	Nutrition, activities of daily living, stress and distress, risk management, medication availability and cost, non-drug treatments, CST, impact of caring, negative aspects of caring, carer needs, services available, signposting information

### Stage 2: Development of the international version

The presentation and accompanying Course Guide were initially developed in four iterations by three researchers who were based in the UK, India and Brazil. Examples of changes between iterations included moving information from slides to the Course Guide to ensure that the presentation was not too text intensive and the addition of standardised handouts as appendices in the Course Guide. The Course Guide contained a loose script that presenters were asked to familiarise themselves with and headings referred to specific slides in the presentation. Descriptions of activities were placed in activity boxes throughout the Course Guide and the handout needed for each activity was highlighted in purple.

Versions four of both the Course Guide and presentation were then circulated amongst the CST-International team for feedback, of which six people responded (one UK-based consultant geriatrician and one professor of clinical psychology, two Brazil-based psychologists, one India-based psychiatrist and one Tanzania-based occupational therapist). Feedback at this stage indicated concern that information on the pathology of Alzheimer’s disease and other dementias in the presentation may be too advanced for a carer audience and that concepts such as the biopsychosocial model of dementia may be too complex to translate adequately. This part was subsequently simplified and condensed. Furthermore, slides explaining the biopsychosocial model were disaggregated to include definitions and examples for each component. Some alternatives were provided in the guide targeting audiences with different levels of knowledge and background. For example, a condensed explanation for the types of dementia was accompanied by an alternative detailed explanation as well so that the presenter can choose an appropriate explanation depending on the audience and their ability. This was essential to increase the flexibility for delivering the course to different groups of people while also maintaining consistency across sites.

After addressing this feedback version five of the presentation and Course Guide were finalised. The Course Guide consisted of seven sections (Table 3) and the presentation contained 75 slides. This version was termed the DAC-International and acted as a template for local version development. The DAC-International will be made freely available online at a later date.

**Table 3.** Content of the International Dementia Awareness Course Guide (V5).

Section	Description
Overview	Instructions on how the course should be run and for making local amendments
Welcome	Introduction, purpose, confidentiality and peer learning
Module 1: What is dementia?	Pathology, prevalence and models
Module 2: Positive engagement	Psychological concepts and activities for positive communication and engagement
Module 3: Caring for someone with dementia	Practical information on caring, services and the effects of caring
Handouts	Case studies, positive person work, behavioural charts, activities of daily living assessment
Signposting	All information on services replicated and given to caregivers as a resource

### Stage 3: Development of local versions

To ensure that local adaptations were made consistently, highlighted text was placed at appropriate points in both the Course Guide and presentation for the DAC-International indicating where these should be made (Table 4). DAC-Brazil was translated into Portuguese and DAC-Tanzania was translated into Swahili. Due to the diversity in India, DAC-India was developed as four separate courses (DAC-Chennai, DAC-Mysore, DAC-Thrissur and DAC-Delhi) in four languages (Tamil, Kannada, Malayalam and Hindi, respectively).

**Table 4.** Adaptations and Amendments to the Course Guide for local sites.

Section/Module	Slide	Amendment Requested
Welcome	1: Title Slide	Speakers to add their name and profession to the title slide.
What is dementia?	7: Dementia in …	Add country specific graphs or pictures to explain prevalence. Emphasis should be on the fact that caregivers are not alone, and it is a growing public health problem.
	8 – 10: Dementia myths (Activity)	Add common misconceptions about dementia that are specific to your country.
	Side 13 – 17: Making a cup of tea (Activity)	This activity can be amended for any other common daily activity that requires a series of steps and conducive environment to complete.
Positive Engagement	Slide 23-29: Malignant Social Psychology	Commonly seen behaviours are elaborated in slides that follow. If there are other behaviours that are more commonly seen in the country where this is being delivered, you can use these instead.
	Slide 30 – 33: Case Study (Activity)	The case study can be modified to suit the cultural context of your country (e.g. names, nurse replaced by informal caregiver like a spouse or son, changing the setting from a care home to the persons own home etc.)
	Slide 43 – 44: Exercising the brain	When someone has dementia, people often take over their responsibilities regardless of whether the person can or can’t do it [give country specific examples here]. Whilst they may not be able to do activities independently, you could ask for their help and involve them in their previous responsibilities [give country specific examples here].
	Slide 45: New thoughts, ideas and associations (Activity)	Famous faces that are specific to your setting should be added.
	Slide 47: Promoting strengths and mitigating weaknesses	Briefly go over **sensitive orientation** (orienting person to reality in subtle and dignified ways – as opposed to testing questions or patronizing approaches- provide culture specific example) and **cues for recall** (sometimes it is better to provide information that you feel the person might struggle with in subtle ways instead of repeatedly asking them – provide culture specific example) in this slide.
Caring for someone with dementia	Slide 52: Building and strengthening relationships	Culture specific examples of possible events/spaces that could help socialize the person with dementia e.g. market days/village events should be added.
	Slide 56: Stress and distress	Physical pain and infections can be hard to communicate for a person with dementia, so you should see a doctor or professional if you think they may be in pain. Presenters may wish to add the local procedure here.
	Slide 57: Risk management	Safe walking is allowing the person with dementia to travel outside alone but putting in place safety measures. These examples can be swapped for each country like tags with their address and contact information, or equipping them with GPS trackers.
	Slide 60: Medication and cost	This section should be amended and must include information on the following for each country:
		- Medication available in country
		- Cost/insurance coverage
		- How caregivers can access this medication.
	Slide 61-62: Non drug treatments	This should be amended to include services that provide non-drug intervention for dementia in your country, how caregivers can access this and any contact information e.g. accessing Occupational Therapy services or home care services. An additional blank slide is included for further information
	Slide 69 – 70: Caregiver needs	Amended to include practical information and referral information that caregivers can use to meet the below needs:
		- Need for information (on dementia and caregiving, expert guidance etc.)
		- Need for emotional support (to manage and improve emotional wellbeing)
		- Need for normative support/validation (‘I am doing the right thing right?’)
		- Need for physical support (with assisting Activities of Daily Living (ADL) etc.)
	Slide 71: Services for people with dementia	Amend this slide to include practical information on services that people with dementia can access. Examples of potential interventions are included on the slide.
	Slide 72: Signposting	This slide should include information on available support services for caregivers in your country/region (possibly in the listed topics) to which they can be sign posted for additional help.

#### Dementia Awareness Course – Brazil (DAC-Brazil)

This was initially developed by a team of researchers in both Rio de Janeiro and São Paulo, in order to develop a unique Brazilian version but with distinct signposting information for each site. This decision was made considering that, overall in Brazil, knowledge of dementia is poor (Amado & Brucki, 2018); thus, general information about dementia was considered important for both sites. However, because of the different resources for people with dementia and their caregivers available locally and the socioeconomic and cultural diversity across the country, signposting information was amended depending on the region. The teams considered it more appropriate to adapt and provide information about non-pharmacological or other types of treatments available, for instance, according to each region.

#### Dementia Awareness Course – India (DAC-India)

The content for the Indian versions did not require major amendments. Signposting information was also modified to be more site specific, similar to the Brazilian versions, and culture specific information was added to make courses more relevant. For example, a common myth that was added to the presentation is that the person with dementia has ‘Bad Karma’, which they can be blamed for or that they have been victims of Black Magic. The rest of the content of the course was retained as they were with minor changes in the form of culture specific names for activities and locally relatable examples to explain various principles.

#### Dementia Awareness Course – Tanzania (DAC-Tanzania)

The DAC-Tanzania was developed by researchers who were located in both Tanzania and the UK. Those from the UK had been involved with dementia research in Tanzania for 10 years. Although in Tanzania there are more than 128 tribes, who speak one or two other languages, culturally they have many things in common and, therefore, one Swahili version was developed. Another consideration was literacy, as some caregivers had not been to school or have achieved only primary education. Materials and language were subsequently simplified so that all information could be followed and understood by everyone in the group. The signposting information was localised, giving information on local hospitals and occupational therapy services.

### Stage 4: Local acceptability and feasibility

#### DAC-Brazil

The first delivery of the course occurred at a caregivers’ association in Rio de Janeiro, with 13 carers from the association attending. The attendees were diverse in terms of experience with dementia and caregiving, as some had been caring for their relatives for several years and others had relatives who received the diagnosis months prior to the course. All the attendees who gave feedback (eight out of 13) found the different modules of the course instructive, giving a broad view of various aspects of dementia and caregiving. Some caregivers also highlighted the relevance of the case studies and practical examples, while others noted the importance of exchanging experiences with the speakers and with the other attendees. The participants did not feel that more information needed to be included, and all stated they would recommend the course. Based on this first delivery of the course, which took just over three hours, the team noticed the need to increase the course duration to allow more time for group discussions and participants’ feedback at the end (changing the initial proposal of three hours to a four hour-course).

#### DAC-India

Seven caregivers attended the first delivery of the course at a non-governmental organisation (NGO) in Chennai, taking place over three hours. Participants were carers of those attending CST groups, and the course was further promoted through social media channels. Five out of seven caregivers provided feedback. The course was delivered in a mixture of Tamil and English to bilingual speakers, but most caregivers preferred the course be delivered in Tamil with English being used more sparingly. They also preferred the slides to be in Tamil to be able to follow along more easily. Attendees found different parts of the course the most helpful, with some engaging particularly in the concept of a biopsychosocial approach to understanding behaviour in dementia. Others found practical advice on caring most helpful and uplifting as it gave the caregivers a sense of hope through realistic changes they can make in their care routines. Other caregivers noted that sections on caregiver wellbeing and learning from their peers during the session were the most helpful. Caregivers expressed that they would have preferred the course to include more case studies and more content on practical ways to manage caregiver distress. All carers found the course informative and beneficial, and would recommend the course to others. In Mysore, Thrissur and Delhi, all courses were finalised, but due to the coronavirus pandemic (COVID-19), teams could not pilot the course in these settings. These courses will instead be piloted at a later date.

#### DAC-Tanzania

In Tanzania, the course was delivered in Moshi, taking place over four hours, and attended by 14 caregivers. A second training session was delivered over four and a half hours to 51 carers in Arusha by a psychologist and a medical doctor. Feedback received from the caregivers was that the training provided them with knowledge which they did not have before about dementia and caring for someone with dementia. Many of the participants found practical information on the management of dementia particularly useful and noted that medication was hard to obtain or too expensive. The majority of carers recommended the course, but some suggested that the course should take place over two days rather than one.

## Discussion

Using an iterative, four stage methodology with input from clinicians and researchers in four countries, a brief and acceptable educational course on dementia for carers was developed. The course consisted of an International template, with standardised instructions and amendments made for each country in which it is used. The template was used to create culturally appropriate versions of the course for use in Brazil, India and Tanzania. Reflecting the diversity of states and different languages spoken in India, four versions of the course in India were needed (Mysore, Chennai, Thrissur and Delhi).

This is, to our knowledge, the first example of an educational intervention for caregivers of people with dementia specifically developed as a template, where additions and adaptations for individual countries are considered from the outset. This consideration of cross-cultural validity resulted in highly standardised methodology, from which culturally appropriate local courses were developed, translated and the acceptability evaluated. Other such courses are usually developed in HICs before undergoing a cross-cultural adaptation and validation by individual teams in each country. However, courses developed in HICs are often underpinned by, or specific to, western cultures (Rathod et al., 2019) and cross-cultural adaptations are often individualistic, on an intervention-by-intervention basis (e.g. Matarazzo et al., 2014; Mkenda et al., 2016; Olsson et al., 2016).

In addition to cross-cultural validity, it is hoped that interventions such as the DAC may increase capacity or provide additional training opportunities for healthcare professionals working in these areas, provided that is deemed effective in the large-scale study. Using a train-the-trainer modality, local teams in Brazil, India and Tanzania have successfully trained those in their immediate teams to deliver the DAC, resulting in both an increase in the capacity of the researchers and wider dissemination of the intervention.

Whilst COVID-19 prevented teams from running the course in Delhi, Mysore and Thrissur, the DAC was received well in Brazil, Chennai (India) and Tanzania, with caregivers noting the usefulness of both practical information and psychological principles. Feedback from the Brazil team indicated that attendees valued the peer-learning nature of the course, where exchanging ideas and suggestions was encouraged. Interventions or services that incorporate peer support have previously been identified as an important means of providing opportunities for caregivers to talk freely about difficult experiences, whilst learning how others had coped with such experiences (Greenwood et al., 2013). Involvement and respect are also key principles of CST, which may contribute to increases in quality of life documented as part of CST (Lobbia et al., 2018).

Feedback also indicated that using psychological principles and theories provided an alternative narrative through which to understand dementia. The use of psychological therapies is a strength of this course and an important means of addressing stereotypes that are reinforced by prevailing medical models. In particular, medical models can perpetuate the stereotype of dementia as an ‘inexorable decline in all aspects of human functioning including loss of personhood and self’ (ADI, 2019, p.18).

## Methodological problems and limitations

Due to varying workloads and responsibilities of the team, there was some disparity amongst the level of input across countries. However, the DAC-International was developed with researchers from the UK, Brazil, India and Tanzania ensuring that generalisability and cross-cultural validity was as high as possible.

The three countries in which the local DACs were developed were diverse, with differing levels of economic development, service provision, attitudes to, knowledge and awareness of dementia. However, they were all designated LMICs and, as such, the acceptability and feasibility of the course has yet to be established in HICs. However, as the development team consisted of a combination of clinicians and researchers based in HICs and LMICs, it is likely to be acceptable to caregivers in both contexts.

Whilst we assessed many aspects of acceptability and feasibility, not all aspects of feasibility were addressed. In some sites recruitment was carried out by third parties, and advertising took place over social media, as is common in many research projects. This method of recruitment does not enable an accurate picture of how many people have information about the study and which proportion decides to participate. However, in further large-scale work participant refusal rate will be collected alongside further feasibility data such as ability to recruit a sufficient sample size.

Originally, four versions of the course were planned for India. However, following the successful delivery in Chennai but prior to the course being run in Mysore, Thrissur and Delhi, COVID-19 reached India, and staff were not able to deliver the course. Other avenues for facilitating the course, such as online delivery, are currently being evaluated by the team and it is hoped that this work will resume shortly.

## Future research

As the evaluation planned as part of this study was a preliminary assessment of acceptability of the course to caregivers, no formal quantitative outcomes measures were used. However, before a large-scale assessment of effectiveness of the course is undertaken, a feasibility assessment with suitable outcomes which avoid Western conceptualisations of the concepts, an assumption of literacy and outcomes that have been subject to a psychometric evaluation in each country must be identified. Work to identify appropriate outcomes is ongoing (Du et al., 2021) but concepts that are likely to be of relevance are carer self-efficacy, sense of competence and attitudes toward people living with dementia. It would also be beneficial to evaluate affiliate stigma to explore whether increasing knowledge has a positive or negative effect on this highly complex concept. The DAC subsequently can be evaluated as a large scale, multi-country randomised controlled trial to thoroughly establish both the cross-cultural reliability of the methodology used to make local versions and the effectiveness of the course. Due to COVID-19 and its particularly adverse effect on older adults, it may also be appropriate to explore the acceptability of delivering the course remotely, in an online format.

The DAC-International acts as a template or standardised methodology for which other countries can develop a country specific DAC. As mentioned, the course has primarily been validated in the LMIC context and, to further assess its generalisability and cross-cultural validity, future studies in other LMIC and HIC contexts are needed. In addition, future research may wish to explore feasibility in both private and public healthcare settings.

The DAC was developed based on the assumption of non-existent or limited prior knowledge of dementia, thus suitable for a lay audience. However, as most of the information presented is regarding psychological concepts or interventions, it is likely the DAC will be of interest to caregivers who have further experience or knowledge of dementia. It may also be useful for support workers who have limited prior knowledge or experience of caring for someone with dementia. This and its impact on people with dementia should be formally evaluated and, if found to have a positive effect, could feasibly be implemented as part of an induction or mandatory training for appropriate healthcare professionals in systems such as the UK National Health Service (NHS).

## Conclusion

With an international team of researchers and clinicians, a standardised methodology for culturally valid brief and acceptable educational courses for caregivers of people living with dementia in LMICs was developed. The developed courses were based on underlying psychological theories and principles and, during an initial acceptability test, feedback from recipients in Brazil, India and Tanzania indicated that it was well received. Further testing to assess feasibility and a large-scale evaluation of efficacy will determine the quantitative effects of the courses.

## Appendix

### List of abbreviations

BPSD: Behavioural and Psychological Symptoms of Dementia
CST: Cognitive Stimulation Therapy
CI: confidence intervals
DAC: Dementia Awareness for Caregivers Course
DAC-International: Dementia Awareness for Caregivers Course – International version
DAC-Brazil: Dementia Awareness for Caregivers Course – Brazil version
DAC-India: Dementia Awareness for Caregivers Course – India version
DAC-Tanzania: Dementia Awareness for Caregivers Course – Tanzania version
HIC: high-income country
LMIC: low- and middle-income country
NHS: National Health Service
NGO: non-governmental Organisation
PPI: patient and public involvement
SMD: standardised mean difference
UK: United Kingdom of Great Britain and Northern Ireland
USA: United States of America
WHO: World Health Organisation
